# Supplementary material for: The interactome of intact mitochondria by cross-linking mass spectrometry provides evidence for coexisting respiratory supercomplexes
Source: Mol Cell Proteomics. 2017 Dec 8;17(2):216–32. doi: 10.1074/mcp.RA117.000470 (PMC5795388; doi:10.1074/mcp.RA117.000470)
Supplement: Supplemental Data [file supp_RA117.000470_133922_0_supp_23978_fzffwf.zip › spectra_annotation/mito_spectra_annotation/426-1-4-1-8-1.pdf]

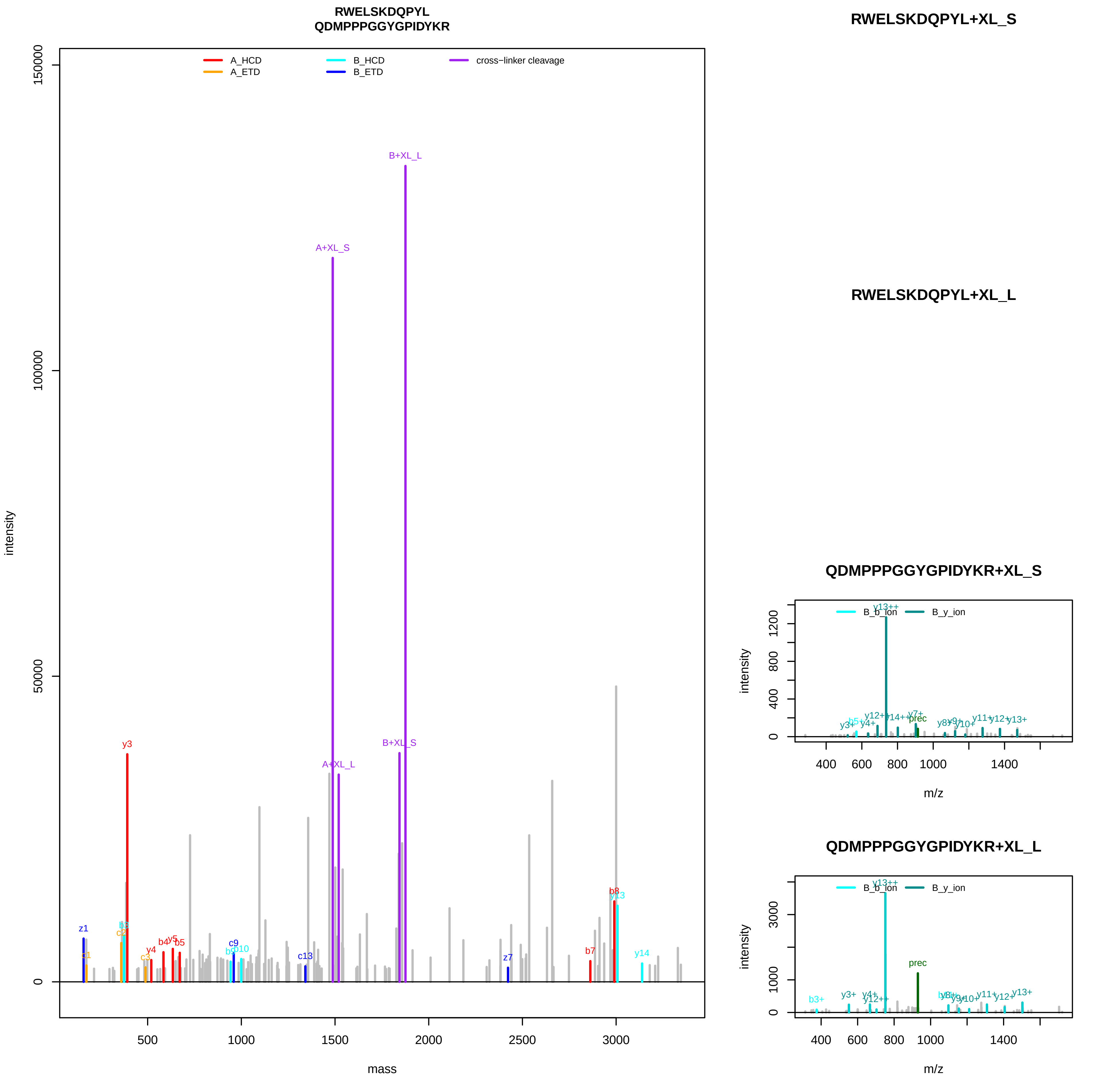

RWELSKDQPYL
QDMPPPGGYGPIDYKR
A_HCD
A_ETD
B_HCD
B_ETD
cross−linker cleavage
intensity
mass
0
50000
100000
150000
500
1000
1500
2000
2500
3000
B+XL_L
A+XL_S
B+XL_S
A+XL_L
y3
z1
c1
c2
c3
y4
b4
y5
b5
c9
c10
c13
z7
b7
b8
y13
y14
RWELSKDQPYL+XL_S
RWELSKDQPYL+XL_L
QDMPPPGGYGPIDYKR+XL_S
B_b_ion
B_y_ion
y13++
prec
y3+
b5+
y4+
y12++
y14++
y7+
y8+
y9+
y10+
y11+
y12+
y13+
intensity
m/z
0
400
800
1200
400
600
800
1000
1400
QDMPPPGGYGPIDYKR+XL_L
B_b_ion
B_y_ion
y13++
prec
b3+
y3+
y4+
y12++
y8+
y9+
y10+
y11+
y12+
y13+
intensity
m/z
0
1000
3000
400
600
800
1000
1400
